# Materia Medica and Therapeutics

**Published:** 1880-10

**Authors:** W.W. Smith

**Affiliations:** Practitioner


### MATERIA MEDICA AND THERAPEUTICS.

The Improbability of any General "Law of Therapeutics."—The desirability of a general law that might introduce simplicity into the realm of therapeutics is not in question. With those who advocate its possibility the wish has been father to the thought. In considering the question, it is natural to turn to physical science and its familiar laws for simple illustrations of the broad conditions necessary to the existence of any natural law. For instance, gases are expanded by heat; water rises to its own level; matter is subject to the law of gravitation; the velocity of a falling body increases in the ratio of the distance that it traverses. Here we have several groups of objects to which laws apply. The individual members of some groups differ widely; thus there are many kinds of gases, and many forms of matter. Nevertheless the members of each group possess certain common characteristics of an essential and definite kind. Gases in general, bodies of water in general, innumerable forms of matter, falling bodies in general, possess such group-characteristics. And the fixed truths called laws which apply to each group, obviously apply to all individual objects possessing those essential common characteristics. Physiological laws, although more complex, illustrate the same broad conditions. For example, the law that reflex movements are excited more slowly through the sympathetic than through the cerebro-spinal system, and continue for a longer time after the removal of the exciting cause. This law is illustrated when we compare the deliberate peristaltic movements of the intestines which are excited by irritation of their sympathetic nerves, with the quick spasmodic action induced by tickling the sole of the foot. Here are definite elements. The practitioner knows certain essential characteristics which belong to each of the two great divisions of the nervous system throughout its extensive distribution. Reflex movements, the exciting cause, time occupied, are each conceptions which differ widely in their details, but about which also certain definite essential characteristics can be predicted as common to each. The proposition can hardly be denied that natural laws apply only to groups of objects or conceptions, the individuals of which groups possess certain definite characteristics which are common to the whole. *A law cannot apply to objects which admit of no such grouping.*

Indefinite words have much to answer for. But for the vague use of the word " disease " practitioners possessed of modern pathological knowledge could hardly be found to maintain the possibility of finding a general " law of cure ; " for *diseases in general appear to admit of no true grouping.* There is indeed one way of regarding them which gives them unity, and is sufficiently simple. Superstition and ignorance have in all ages regarded " Disease " as a peculiar entity, a kind of evil influence which has taken possession of the body. But, when analyzed, diseases are found to consist of complex and varying conditions with no common group-characteristics. Every attempt at a scientific definition embracing diseases in general is necessarily vague. Dr. Green, in his admirable *Introduction to Pathology*, writes :—" By disease is understood some deviation from the state of health ; a deviation consisting for the most part in an alteration in the functions, properties or structure of some tissue or organ owing to which its office in the economy is no longer performed in accordance with the natural standard." Probably it would be difficult to improve on this statement. But it contains no indication of any definite characteristic which is common to diseases in general. "Alteration" is of necessity a diffuse and indefinite word. The occurrence even of this vague " alteration " is variable ; it takes place only " for the most part." Its seat is indefinite, viz.: " some tissue or organ."

The attributes of such tissue or organ which are subject to "alteration" are variable, viz., "its functions, properties, or structure." To obtain a more comprehensive statement it is necessary to resort to a negative description which is still more vague, viz.: "some deviation from the state of health," and this negative description is merely reiterated in referring to "non-accordance with the normal standard." If the able author were asked what is "the state of health," or "the normal standard," he might vary the reply, but could probably assert nothing more definite than that it is the absence of disease—thus adopting the circular method. It would have been unscientific to have attempted anything more positive.

It is true that our knowledge is imperfect. But all that has been learned tends to indicate that it is not owing to imperfect knowledge that we are unable to group diseases in general, or find a comprehensive definition of "disease." The results of incessant pathological and clinical industry throughout civilization tend to separate diseases and to group them into subdivisions, but afford no indication that there is anything distinctive that applies to diseases in general.

Take a few patients, say at a hospital out-patient room. One has a misshapen ineffectual valve in that living pump called the heart, and all the mechanical consequences. He is said to have "valvular disease." Another has serious symptoms due to the fact that a growth detacted from one of the heart's valves has been washed along in the circulation, and now forms a solid plug stopping up the channel of an artery. He is said to have "embolism." Another has one or more thriving worms, with tentacles fixed in his intestinal mucous membrane, or encysted in his liver. Another has a concretion of hard, fatty substance lodged in a tube which is too small to readily admit it, and the living tube is spasmodically contracting upon the intruder to the agony of the patient—he is said to have gall-stones. Another shows a joint, red, swollen, and painful, and is said to have "synovitis." Another has breathed an atmosphere charged with special poison, his blood and all the constituent fluids of his body, perhaps the solids too, have become poisoned and poisonous; he has an infectious fever. Some diseases are essentially due to "coarse" alterations, such as the embolism, whilst simple inflammation, or the growth of a tumor, are essentially connected with delicate microscopic processes. Patients may complain of a variety of symptoms which only by skilled investigation are found to be due possibly to a plug of cerumen in the ear, or a stone in the kidney, or a broken rib. Investigation has progressively removed various diseases from the obscure class, and has found that they depend on causes of a kind which are more or less palpable. It is only within the present century that it has become generally recognized that itch is due to a colony of parasites. Not only is palpable embolism of modern discovery, but the obscure disease chorea is attributed on reliable authority to minute emboli plugging some of the brain's capillaries. Whether "functional disease" has any real existence may well be questioned. Derangement of function probably results in every case from derangement of structure, whether that derangement be coarse and persistent, or whether it be delicate and transitory. As medicine learns more and more of the essential phenomena of diseases, it may be that she will dismiss "functional disease" from her momenclature and regard it as a mere cloak for the ignorance of the past. The diseases of the nervous system which are commonly called functional, are well classified, for instance, by Niemeyer, as "neuroses of unknown anatomical origin." The word "unknown" is a wholesome and refreshing acknowledgment of ignorance, a salutary improvement on the fine expression "functional." The sight of it sends us back to school, and stimulates inquiry.

Only by shutting the eyes to facts, and referring diseases such as the above to one comprehensive group, does it appear possible to maintain the existence of a general law of nature for their remedy. But if they belong to no common group they can possess no common law. At the present time vendors of patent pills and charlatans find it necessary for their purpose to make comprehensive assertions of the unity of disease. All diseases arise from impurity of the blood, their nostrum corrects this, and is therefore a universal remedy. Or all diseases arise from failure of nervous power. Phosphorus or electricity, according to the particular view of the advertiser, is therefore a universal remedy.

To maintain the existence of a universal law of therapeutics appears nearly as unreasonable as if a watchmaker were to advocate a single principle of dealing with the derangements of a watch. The watch may be suffering from congenital imperfections, or on the other hand from senile degeneration, resulting in organic defects; it may be deranged by the presence of foreign bodies, such as dust, or by general injuries, such as a concussion. Paralysis from local injury, such as a fractured spring, must be distinguished from "functional" paralysis, the result of not being wound up. Sometimes it is a question of "humoral pathology" and oil is required. Sometimes there is derangement connected with undue "oxidation" from climatic vicissitudes.

Now a watchmaker fond of generalizations might possibly regard all watch diseases from a comprehensive point of view. He would imitate the medical writers who theorize about "disease." Naturally he would seek a common "law of cure," or having already advertised the "law of cure," he might not improbably theorize about "disease" in watches, to explain its *modus operandi.*

Had Hahnemann contemplated the facts of modern morbid anatomy and pathology, it is doubtful whether he would have written such a sentence as this:—"To effect a mild, rapid, certain, and permanent cure, choose *in every case of disease,* a medicine which can itself produce an affection similar to that sought to be cured." Practitioners seem unconsciously clinging to mediæval traditions when they talk of the possibility of a natural common "law of cure." So long as an entity, "disease," is contemplated, the idea is tenable enough. But the bogey belongs to polytheism. Its ancient adherents associated the medical practitioner with magicians, astrologers, and wizards. His duty was by the combined use of drugs, rites, and incantations, to charm, coax, or frighten away the mysterious evil thing that had taken its abode in the unfortunate patient. The "medicine man" of savages has similar duties. And the kind of phraseology commonly dealt in by the best exponents of a supposed comprehensive law of therapeutics is significant. It indicates how impossible it is to maintain their doctrine except by theorizing about the imaginary entity—" disease." Such language, and the ideas associated therewith, tend toward the arrest and retrogression of medical knowledge, just as the superstition of the Middle Ages brought all science to a standstill.—W. W. SMITH, *Practitioner.*

